# Predictive role of diffusion‐weighted whole‐body MRI (DW‐MRI) imaging response according to MY‐RADS criteria after autologous stem cell transplantation in patients with multiple myeloma and combined evaluation with MRD assessment by flow cytometry

**DOI:** 10.1002/cam4.4136

**Published:** 2021-07-15

**Authors:** Angelo Belotti, Rossella Ribolla, Valeria Cancelli, Alberta Villanacci, Valentina Angelini, Marco Chiarini, Viviana Giustini, Giulia V. Facchetti, Aldo M. Roccaro, Samantha Ferrari, Annalisa Peli, Chiara Bottelli, Chiara Cattaneo, Claudia Crippa, Monica Micilotta, Barbara Frittoli, Luigi Grazioli, Giuseppe Rossi, Alessandra Tucci

**Affiliations:** ^1^ Department of Hematology ASST Spedali Civili di Brescia Brescia Italy; ^2^ Department of Radiology ASST Spedali Civili di Brescia Brescia Italy; ^3^ Clinical Chemistry Laboratory Diagnostic Department ASST Spedali Civili di Brescia Brescia Italy; ^4^ Clinical Research Development and Phase I Unit ASST Spedali Civili di Brescia Brescia Italy

**Keywords:** diffusion‐weighted whole‐body MRI, minimal residual disease, MRD, myeloma, MY‐RADS, transplant

## Abstract

**Background:**

Diffusion‐weighted whole‐body MRI (DW‐MRI) is increasingly used in the management of multiple myeloma (MM) patients, but data regarding the prognostic role of DW‐MRI imaging response after treatment are lacking. The Myeloma Response Assessment and Diagnosis System (MY‐RADS) imaging recommendations recently proposed the criteria for response assessment category (RAC) with a 5‐point scale in order to standardize response assessment after therapy, but this score still needs to be validated.

**Methods:**

We investigated the prognostic role of RAC criteria in 64 newly diagnosed MM patients after autologous stem cell transplantation (ASCT), and we combined the results of MY‐RADS with those of minimal residual disease (MRD) assessment by multiparametric flow cytometry (MFC).

**Results:**

Superior post‐ASCT PFS and OS were observed in patients with complete imaging response (RAC1), with respect to patients with imaging residual disease (RAC≥2): median PFS not reached (NR) versus 26.5 months, *p *= 0.0047, HR 0.28 (95% CI: 0.12–0.68); 3‐year post‐ASCT OS 92% versus 69% for RAC1 versus RAC ≥2, respectively, *p* = 0.047, HR 0.24 (95% CI: 0.06–0.99). Combining MRD and imaging improved prediction of outcome, with double‐negative and double‐positive features defining groups with excellent and dismal PFS, respectively (PFS NR vs. 10.6 months); *p* = 0.001, HR 0.07 (95%CI: 0.01–0.36).

**Conclusion:**

The present study supports the applicability of MY‐RADS recommendations after ASCT; RAC criteria were able to independently stratify patients and to better predict their prognosis and the combined use of DW‐MRI with MFC allowed a more precise evaluation of MRD.

## Graphical abstract

The present study supports both the applicability and the clinical usefulness of the proposed Myeloma Response Assessment and Diagnosis System (MY‐RADS) recommendations applied to DW‐MRI imaging obtained after first‐line treatment of MM including ASCT. MY‐RADS RAC criteria were able to independently stratify patients and to better predict their outcome and their prognosis. DW‐MRI proved to be a powerful tool for the assessment of residual bone disease by functional imaging: it complemented bone marrow flow cytometry and may, therefore, represent a step forward in the clinical management of the increasing number of MM patients achieving CR with the novel treatment approaches.

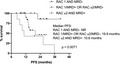

## 1 INTRODUCTION

Significant improvements in the treatment of multiple myeloma (MM) patients have been observed in recent years, thanks to the availability of novel and effective combination therapies with increased rates of high‐quality responses. The development of multiparametric flow cytometry (MFC) and next‐generation sequencing (NGS) diagnostic tools allows the detection of increasingly lower levels of disease inside the bone marrow (BM), strengthening the definition of complete response.^1^ This is particularly relevant for transplant‐eligible MM patients due to the established prognostic impact of MRD negativity after ASCT. Likewise, with the increasing availability of functional imaging techniques, the precise evaluation of response has become feasible also for MM lesions in bones and other organs, as observed in PET/CT studies,^2^, ^3^, ^4^ allowing the combined evaluation of residual MM disease in bones and in BM. Particularly, the high spatial genomic heterogeneity that characterizes MM patients may explain the persistence of residual disease within and outside of the BM in patients with biological complete response: the existence of distinct disease clones within a patient with different genomic profiles underlies the natural history of a disease in which relapses systematically occur.^5^ Therefore, the combined evaluation of marrow MRD and functional imaging assessment is required to examine MM complete eradication. Diffusion‐weighted whole‐body magnetic resonance imaging (DW‐MRI) is a highly sensitive functional imaging technique, but its potential prognostic role in the detection of residual disease after treatment in MM still needs to be established. The differences in the motion of water at a cellular level are the basis of the contrast between tissues observed in the images produced by DW‐MRI; it, therefore, permits to estimate cell density with the so‐called apparent diffusion coefficient (ADC).^6^ Regarding the assessment of imaging response after therapy, the major advantage of this technique is the possibility to improve the morphological response evaluation criteria adopted by radiologists in solid tumors (RECIST criteria)^7^ represented by features as a variation of the dimension of target lesions, further adding functional information (diffusion‐weighted imaging) thanks to the evaluation of water molecules motion within tissues. Indeed, starting from the assumption that the more the tissue is celled, the less will be the movement of water molecules, this translates into the increase of signal DWI sequence (compared to the surrounding background) and the reduction in ADC value that represents the quantitative value of this movement. With the intent to promote standardization in the acquisition, reporting, and interpretation of whole‐body DW‐MRI, the Myeloma Response Assessment and Diagnosis System (MY‐RADS) imaging recommendations have been recently published.^8^ Criteria for response assessment category (RAC) have been proposed with a 5‐point scale defining the probability of complete imaging response (i.e., RAC 1) or progressive disease after therapy (i.e., RAC 5). The assignment of RAC is based on specific morphological findings and quantitative calculation of the ADC after treatment. We performed an external validation of RAC criteria according to MY‐RADS in newly diagnosed MM patients in order to evaluate the prognostic role of DW‐MRI after autologous stem cell transplantation (ASCT). Moreover, in patients with MRD evaluations before maintenance, an exploratory analysis was performed in order to correlate the results of MY‐RADS with those of post‐ASCT MRD assessment by 8‐color multiparametric flow cytometry (MFC, sensitivity 10^−5^).

## 2 METHODS

The outcome of 64 transplant eligible MM patients diagnosed between January 2016 and January 2020 was retrospectively analyzed: patients received ASCT intensification after induction treatment and underwent DW‐MRI evaluation to assess the response at day +100 after transplant. *Subsequent lenalidomide maintenance was started after regulatory approval in Italy (2018) in 48 patients*. Images revision was performed jointly by four radiologists of our institution who were blinded to clinical and biological data. Post‐ASCT progression‐free survival (PFS) was calculated from the date of the last ASCT (the second one in case of double ASCT) until progression or death from any cause. Post‐ASCT overall survival (OS) was calculated until death from any cause or last follow‐up. The predictive role of DW‐MRI response on PFS and OS was analyzed *(in patients receiving double ASCT, DW‐MRI performed after the second one was considered for the analysis)*. Statistical analyses were performed using GraphPad Prism software: Fisher's exact test was performed to compare frequency distributions of the subgroup of patients according to the RAC score. Log‐rank (Mantel–Cox) test applied to the Kaplan–Meyer method was used to estimate survival curves. Cohen's kappa statistics were used to express the level of agreement between MRD and DW‐MRI concordance results. The influence of prognostic factors on outcome was compared by performing the multivariate analysis (Cox proportional hazard models).

### 2.1 DW‐MRI

The protocol includes scanning from vertex to toes in 4–5 slabs depending on patient height, with the axial acquisition of morphological sequences as T1 DIXON and T2 HASTE images, T1 tse and STIR sagittal images on the whole spine and functional axial DWI/ADC images. Total imaging time for the study is about 40 min and no contrast media is administered. Lesions are classified as restricting (low ADC values and signal increase in DWI) and non‐restricting (high ADC values and decrease signal in DWI): a target lesion is defined as a lesion greater than 5 mm identified on T1 and T2 weighted morphological sequences, with high signal intensity on high b‐value images (900 s/mm^2^) and ADC values lower than 1400 μm^2^/sec. When multiple lesions were identified, the final attribution of RAC was referred to the main five target lesions. When assessing response among multiple target lesions, the predominant one (the higher category) was reported for the assignment of the final RAC score. Particularly, the increase in ADC above the cut‐off value of 1400 μm^2^/s has been adopted for the assignment of RAC1. However, ADC values can be influenced by the relative proportion of plasma cells, fat, and myeloid cells within the BM. In order to increase specificity and reduce the risk of false‐positive interpretations, the use of the DIXON fat fraction (FF) sequence allows to evaluate the degree of adipose re‐population of the responding lesions after treatment, therefore adding the ability to discriminate responding lesions compared to lesions resistant to therapy and reducing the false‐positive cases in DWI. Not least, FF sequences obtained by DIXON sequences and associated with DWI/ADC can improve the detection of persistent disease. Diffuse infiltration disease has been also evaluated and suspected in cases with diffuse decreased signal on T1 images, and diffuse increased DWI and STIR skeletal values: the disappearance or appearance of diffuse myelomatous infiltration is part of the morphological findings that define the RAC score. ADC values can change over time and the optimal timing of DW‐MRI after treatment has not been established so far: in fact, an early increase in ADC values is typically observed in responding lesions at 4–6 weeks due to cell death, followed by a decrease together with fatty bone marrow reconstitution.^9^, ^10^ The direction of ADC change and cut‐off values may be therefore influenced by the timing of measurement. As per clinical practice at our institution, we combine the evaluation of response after ASCT according to IMWG criteria by assessing BM at day +100 before starting maintenance therapy. We elected to perform DW‐MRI at the same timepoint, in order to reduce time‐related variability and to enable a correct imaging assessment.

### 2.2 MRD assessment by MFC

MFC analyses were performed with FACSCanto II flow cytometer equipped with FACSDiva software (BD Biosciences) on marrow aspirates before maintenance therapy and sample processing was done within 24 h after collection using either of two tubes with eight colors. According to consensus recommendations,^11^, ^12^ a minimum of 1 × 10^6^ events for each sample was acquired, defining MRD‐negativity as the detection of <50 clonal plasma cells among ≥500,000 nucleated cells (sensitivity level from 10^−4^ to 10^−5^; threshold =0.01% of nucleated cells).

## 3 RESULTS

**FIGURE 1:**
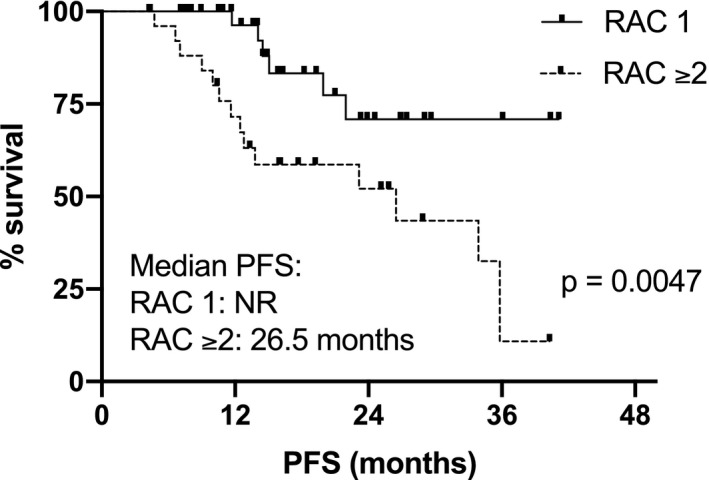
Post‐ASCT PFS according to RAC (RAC 1 vs. RAC ≥2)

**FIGURE 2:**
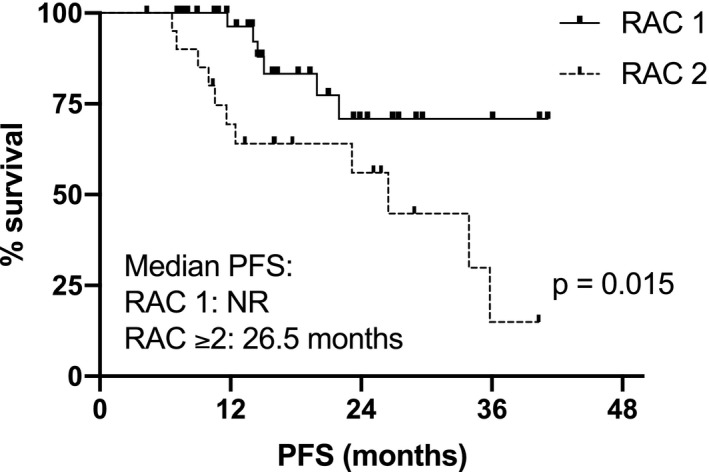
Post‐ASCT PFS according to RAC (RAC 1 vs. RAC 2)

**FIGURE 3:**
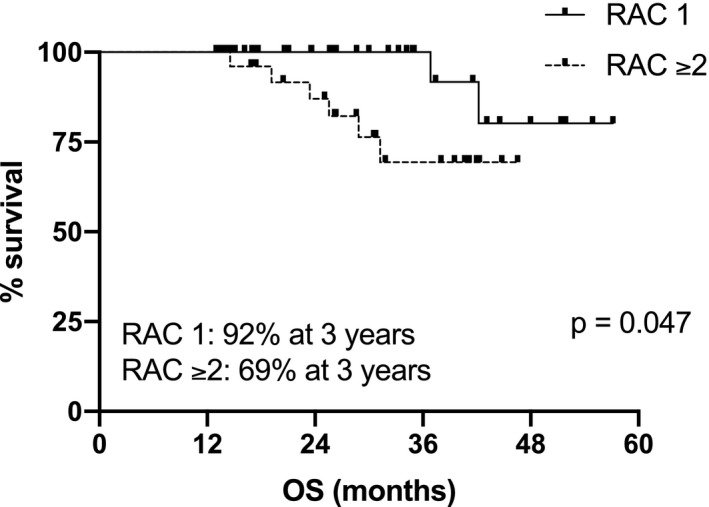
Post‐ASCT OS according to RAC

**FIGURE 4:**
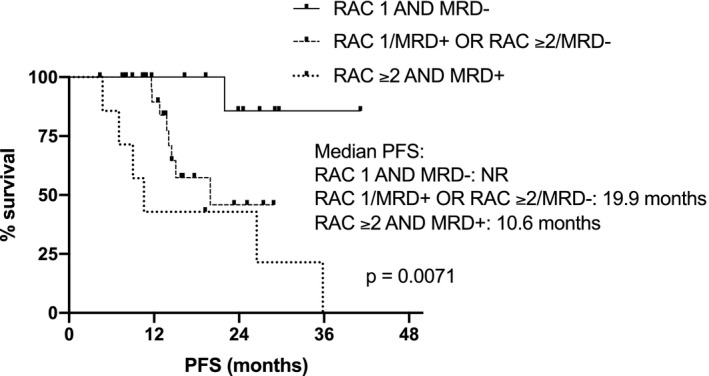
Post‐ASCT PFS according to DW‐MRI response and MRD

The following induction regimens had been used in the 64 patients studied: bortezomib, thalidomide, dexamethasone (VTD) in 53 (83%), bortezomib, lenalidomide, dexamethasone (VRD) in 5 (7%), daratumumab, bortezomib, lenalidomide, dexamethasone (Dara‐VRD) in 4 (6%), carfilzomib, lenalidomide, dexamethasone (KRD) in 1 (2%), bortezomib, cyclophosphamide, dexamethasone (VCD) in 1 (2%). 41 patients (64%) received single ASCT (MEL200 mg/sqm conditioning), whereas double ASCT was performed in 23 patients (36%). Median follow‐up was 29 months from diagnosis and 19 months from ASCT; patients’ characteristics are shown in Table 1. International Myeloma Working Group (IMWG) responses were: 10 PR (16%), 15 VGPR (23%), 30 CR (47%), and 9 sCR (14%). Complete imaging response after ASCT according to MY‐RADS was achieved in 37 patients (RAC 1: 58%), whereas residual disease was observed in 27 patients (RAC ≥2: 42%; RAC 2: 22 (34%), RAC 3: 4 (6%), RAC 4: 1 (2%), respectively). Agreement among radiologists in the assignment of RAC score was high. Discrepancies in the initial classification occurred in two patients who were classified as RAC2 and RAC4, respectively, after joint discussion. No correspondence was observed between the depth of response according to the IMWG criteria and the distribution of patients with residual imaging disease across the different response levels, as well as the distribution of patients with high‐risk cytogenetic profile or the number of ASCT performed (Table 2). Patients with RAC 1 achieved significantly higher post ASCT PFS, respect to patients with RAC ≥2: median not reached (NR) vs 26,5 months, *p* = 0.0047, HR 0.28 (95% CI: 0.12–0.68) (Figure 1). Similarly, different survival curves were observed comparing only the subgroups of patients with RAC 1 versus RAC 2 imaging (Figure 2): median NR versus 26.5 months, *p* = 0.015, HR 0.29 (95% CI: 0.11–0.79). Patients with imaging residual disease have shown inferior post‐ASCT OS (Figure 3): the 3‐year post‐ASCT survival rate was 92% versus 69% for RAC1 versus RAC ≥2, respectively, *p* = 0.047, HR 0.24 (95% CI: 0.06–0.99). Among patients with imaging residual disease (27), 16 patients received maintenance therapy whereas 11 did not. With the limit of the low numbers of patients for comparison, no significant difference was found in post‐ASCT PFS according to maintenance versus no maintenance therapy in DW‐MRI positive patients (26.4 vs. 23.2 months, *p* = 0.81). MRD assessment before maintenance with MFC was available in 46 patients and was negative in 25 (54%) of them (9 patients in sCR and 16 CR according to IMWG response criteria). Post‐ASCT PFS was significantly longer in MRD negative versus MRD positive patients: median NR versus 19.9 months, *p* = 0.014, HR 0.28 (95% CI: 0.09–0.78). Comparable OS was observed at the time of follow‐up analysis cut‐off: 3‐year post‐ASCT survival rate was 88% versus 86% (p: NS) in MRD negative and positive patients, respectively. Low concordance was observed between DW‐MRI and marrow MRD results (52%, kappa 0.017: 17% both positive, 35% both negative). According to IMWG, sCR or CR was achieved in 30 of the 46 patients with combined bone marrow and imaging evaluation. Residual disease was identified in 15 of them (50%): in 8 by DW‐MRI only (of note, 2 patients were in sCR), in 4 by MFC only, and in 3 by both methods. Combining the two techniques of residual disease assessment, three subgroups of patients were identified (RAC1 and MRD negative vs. RAC ≥2 and MRD+vs. RAC1/MRD+or RAC ≥2/MRD negative) with a significantly different outcome (*p* = 0.0071). Particularly, significantly superior post‐ASCT PFS was observed in patients with RAC1 and MRD negativity before maintenance, with respect to patients with RAC ≥2 and MRD positive results (PFS NR vs. 10.6monts; *p* = 0.001, HR 0.07–95%CI: 0.01–0.36). Patients with either imaging or MRD positive results have shown intermediate post‐ASCT PFS (median 19.9 months), which was significantly lower with respect to the RAC1/MRD negative group (*p* = 0.037, HR 0.24–95% CI: 0.06–0.91), as shown in Figure 4. No differences were found comparing the subgroup of RAC1/MRD+patients with RAC ≥2/MRD negative patients (data not shown due to small sample size). Among prognostic parameters available in all 64 patients (IMWG response, RAC score, cytogenetic risk profile), multivariate analysis showed that post‐ASCT PFS was independently affected by both high‐risk cytogenetic profile and DW‐MRI persistent disease (RAC ≥2) after transplant, (*p* = 0.048 and *p* = 0.011, Table 3).

**TABLE 1:** Patient characteristics (patients with high risk cytogenetics had t(4;14), t(14;16) or del17p abnormalities. ISS: International Staging System)

Characteristics	
Patients *N*	64
Median age (range)	65 (34–73)
ISS N (%)	
Stage I	20 (31%)
Stage II	21 (33%)
Stage III	23 (36%)
High risk cytogenetics FISH	14 (22%)

**TABLE 2:** Patients characteristics according to RAC score

	RAC1 (*N* = 37)	RAC≥2 (*N* = 27)	*p* value
IMWG response (*N*)			
sCR (9)	7 (78%)	2 (22%)	0.28
CR (30)	16 (53%)	14 (47%)	0.61
VGPR (15)	7 (47%)	8 (53%)	0.34
PR (10)	7 (70%)	3 (30%)	0.5
Single ASCT (41)	25 (61%)	16 (39%)	0.6
MRD positive (21/46)	13 (62%)	8 (38%)	0.99
High risk cytogenetics (14)	6 (43%)	8 (57%)	0.23
Median post ASCT PFS (months); HR 0.28 (95% CI, 0.12–0.68)	NR	26.5	0.0047
3‐year post ASCT survival rate; HR 0.24 (95% CI, 0.06–0.99)	92%	69%	0.047

**TABLE 3:** Multivariate analysis for post‐ASCT PFS. Data are presented as hazard ratio (HR)

PFS	HR (95%CI)	*p* value
IMWG response: <CR	0.43 (0.17–1.03)	0.06
RAC ≥2	0.29 (0.11–0.75)	0.011
High risk cytogenetic	0.39 (0.15–0.99)	0.048

## 4 DISCUSSION

Many effective agents have become recently available for the treatment of MM making the possibility of achieving a cure also in MM a realistic goal. To this end, the detection of MRD after potentially eradicating treatments is of paramount importance. MM is characterized by the patchy distribution of clonal plasma cells both in marrow and in bone tissue; moreover, the presence of multiple clones with different molecular abnormalities is at the basis of potentially different responses to therapies. Therefore, in addition to MFC and NGS techniques for detecting MRD in marrow, there is a need to properly evaluate the presence of MRD also by functional bone imaging, particularly after potentially eradicating treatments like transplant. Prospective studies have investigated the role of PET/CT in recent years,^2^, ^3^, ^4^ leading to the introduction by the IMWG of the concept of PET/CT negativity after treatment as a criterion to further improve the evaluation of MRD complete response.^13^ The prognostic role of conventional MRI (spine and pelvis) and PET/CT was investigated in the multicenter IMAJEM trial^4^ in 134 patients randomized to receive lenalidomide, bortezomib, and dexamethasone (RVD) with or without ASCT, followed by lenalidomide maintenance. In this trial, MRI performed before maintenance was not predictive of PFS and OS due to a significant number of false‐positive results. Conversely, superior PFS and OS were observed in patients with negative PET/CT, compared to patients with the residual disease before maintenance. The study also supported the complementarity of PET/CT and marrow MFC in the prediction of outcome: the concordance between the two methods was low, as confirmed more recently in the CASSIOPEIA study,^14^ again confirming the importance of combining BM and functional imaging assessments for a complete evaluation of residual disease in MM. Similar results were observed in the PET/CT substudy of the FORTE trial,^3^ where concordance between PET/CT and MFC and NGS was higher in the detection of BM residual disease than of residual focal lesions (FL) outside the BM. In parallel with PET/CT, the potential role of DW‐MRI for MRD detection was investigated by Rasche et al.,^2^ where MFC, PET/CT, and DW‐MRI were performed in 168 patients achieving CR after first‐line or salvage treatment. In this study, DW‐MRI detected more patients with residual FL than PET/CT (21% vs. 6%, respectively). Again, the study highlighted that the best PFS outcome was achieved by those patients showing both MFC and imaging negativization after treatment, as compared to patients achieving disease eradication only within or outside the BM. Establishing criteria for a correct interpretation of functional imaging results after induction treatment and transplant to allow standardization of the definition of complete imaging response is an area of active investigation. Indeed, robust data emerged over the last 10 years have proven the prognostic information of PET response before maintenance in several prospective studies.^3^, ^4^, ^15^, ^16^, ^17^ To this end, results of standardization of PET/CT according to Deauville criteria for the metabolic complete response have been recently published.^18^ Similarly, the need for standardization in WB‐MRI reporting of response after treatment resulted in the recently published MY‐RADS guidelines and in the 5‐point scale RAC score.^8^ Such guidelines still require validation within clinical trials, including the assessment of reproducibility. In the present study, MY‐RADS criteria were applied to a population of consecutive MM patients undergoing ASCT as first‐line therapy. Results highlight the ability of DW‐MRI to identify a significant proportion of patients with imaging residual disease after treatment, even among those defined in sCR/CR according to the IMWG criteria (22% of sCR and 47% of CR patients, respectively). Moreover, DW‐MRI detected imaging residual disease also in 20% of MFC‐MRD negative patients. Radiologists did not report difficulties in applying MY‐RADS guidelines and assignment to the different RAC categories was straightforward in the vast majority of cases. The clinical importance of our findings is obvious considering the poor outcome observed in patients with imaging residual disease who had formally achieved a response according to IMWG criteria, whose post‐ASCT median PFS was 26.5 months and 3‐year survival rate of 69%. While the long‐term analysis of the study by Cavo et al.^19^ using VTD followed by single or double ASCT showed a median PFS of 38 or 47 months and a 3‐year survival rate of approximately more than 75%. Of note, 16 patients in our cohort did not receive lenalidomide maintenance therapy because it was not yet approved in Italy at the time of ASCT. Interestingly, with the limit of the low number of cases, the outcome of patients with positive DW‐MRI after transplant seems not to be influenced by lenalidomide maintenance therapy, highlighting the unfavorable prognostic factor of DW‐MRI imaging residual disease after transplant. Hence, this retrospective external validation of MY‐RADS criteria promotes the standardization in reporting functional imaging response after treatment, supporting the ability of DW‐MRI to independently stratify patients with different survival. Data further emphasize the potential use of DW‐MRI as a tool to identify patients at high risk of early relapse after receiving first‐line treatment including ASCT. As already shown in studies comparing the detection of residual disease by PET‐CT or MFC techniques,^2^, ^14^ the low concordance observed in our work between DW‐MRI response and MFC results confirms the complementarity of these two techniques and support their combined use for a correct assessment of residual disease and evaluation of prognosis after transplant. To our knowledge, this is the first report supporting the applicability and the usefulness of the proposed MY‐RADS response criteria in clinical practice. The retrospective nature of our observations and the relatively low number of patients represent limitations of our study, requiring further confirmation in larger prospective multicentric studies. Likewise, statistical significance regarding the relationship between DW‐MRI response and variables such as the number of ASCT performed and high cytogenetic risk could be affected by the small sample size of each subgroup. Also, the evaluation of the inter‐observer agreement between radiologists in reporting imaging responses needs to be verified even though the availability of objective parameters such as ADC values and FF for the classification of imaging results should limit the possibility of disagreement. On the basis of our experience, the application of RAC criteria was not time‐consuming for radiologists and clearly helped in the evaluation of response after treatment, particularly in case of equivocal residual lesions after therapy. In summary, the present study supports both the applicability in real life and the clinical usefulness of the proposed MY‐RADS recommendations applied to DW‐MRI imaging obtained after first‐line treatment of MM including ASCT. MY‐RADS RAC criteria were able to independently stratify patients and to better predict their outcome and their prognosis. Like PET/CT, DW‐MRI proved to be a powerful tool for the assessment of residual bone disease by functional imaging. It complemented bone marrow flow cytometry allowing a more precise evaluation of MRD. It may, therefore, represent a step forward in the clinical management of the increasing number of MM patients achieving CR with novel treatment approaches. Efforts in the standardization of imaging definitions and interpretation of residual lesions could promote in the near future the incorporation of functional imaging in MRD‐driven prospective trials.

## CONFLICT OF INTEREST

Authors declare no conflict of interest to disclose.

## ETHICS STATEMENTS

This study has been approved at our institution by the local Ethics Committee and has been done in accordance with the Declaration of Helsinki.

## Data Availability

The data supporting the findings of this work are not publicly available due to ethical restrictions (available on request from the corresponding author).
